# Supplementary material for: A De novo Peptide from a High Throughput Peptide Library Blocks Myosin A -MTIP Complex Formation in Plasmodium falciparum
Source: Int J Mol Sci. 2020 Aug 26;21(17):6158. doi: 10.3390/ijms21176158 (PMC7503848; doi:10.3390/ijms21176158)
Supplement: Supplementary file 1 [file ijms-21-06158-s001.pdf]

Figure S1:

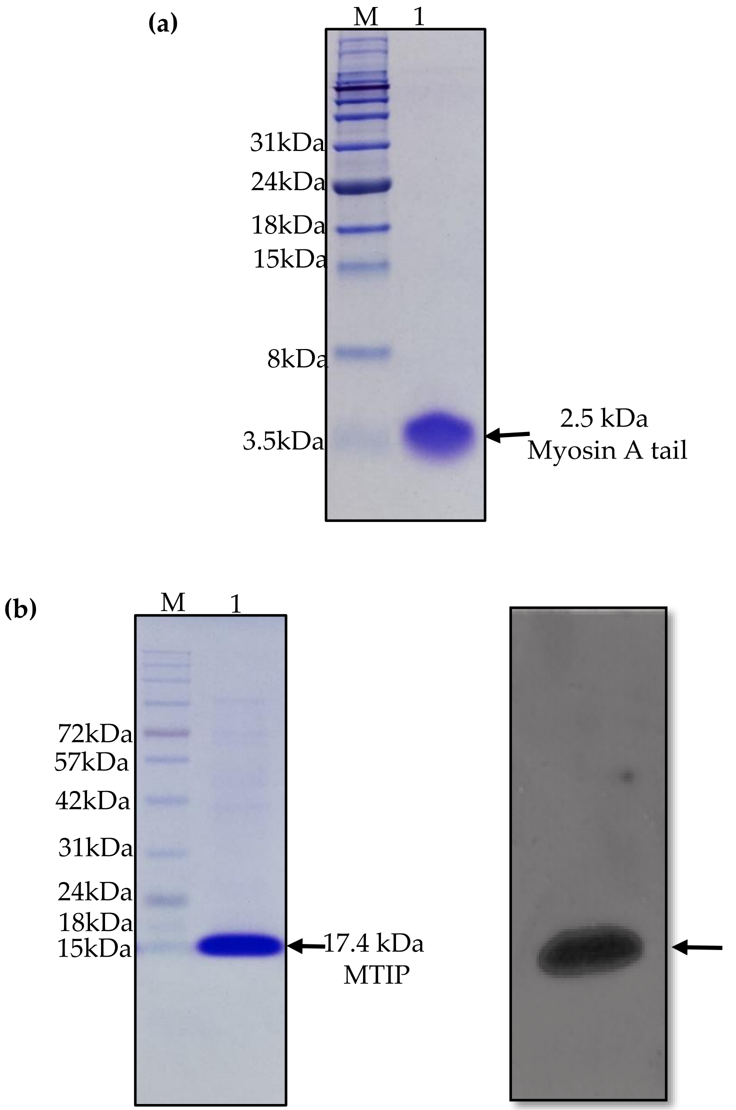

Figure S2:

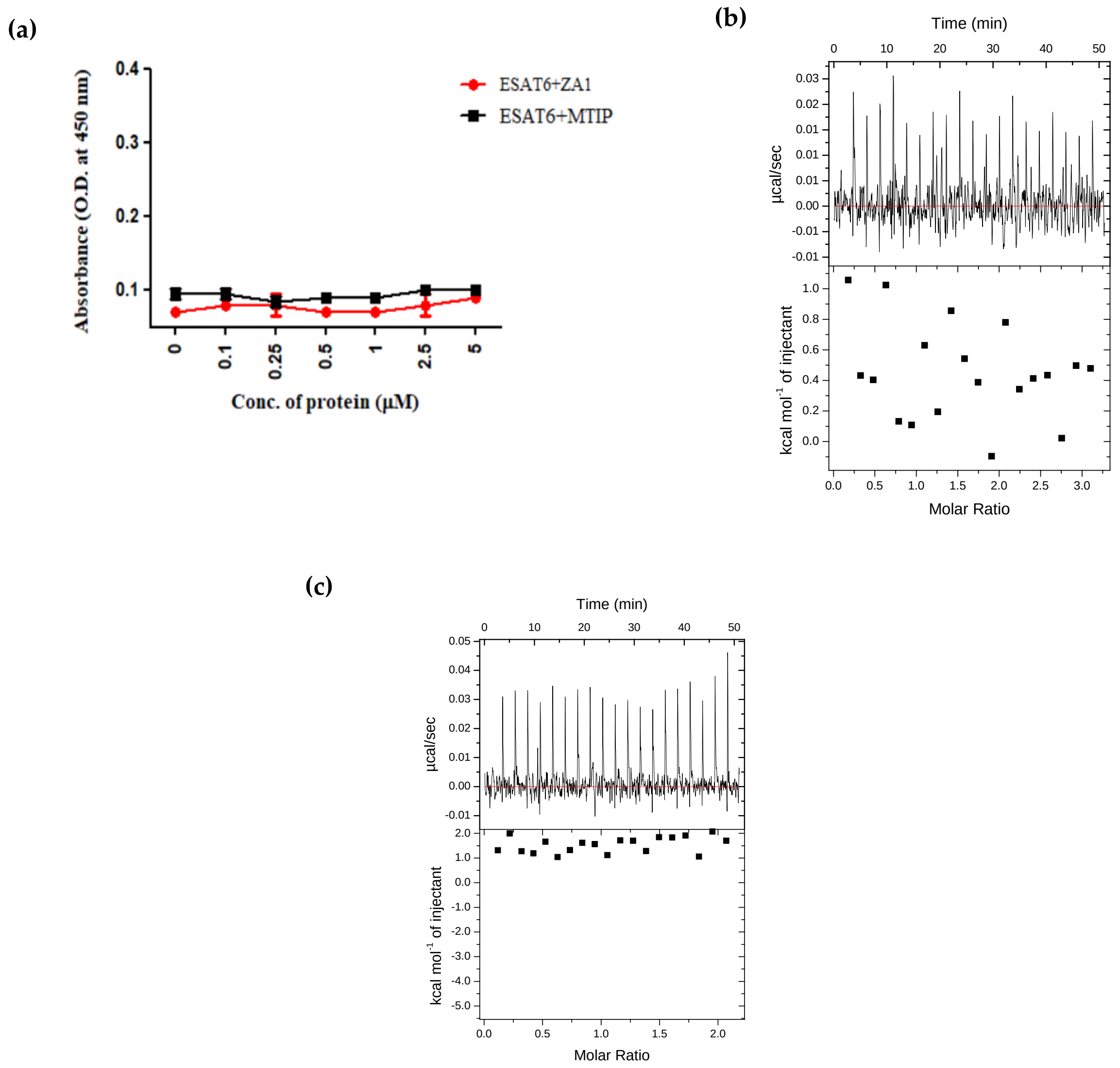

Figure S1: Myosin A tail and expression of MTIP. (a) Synthesized Myosin A tail peptide (2.5 kDa) resolved on Tris-tricine polyacrylamide gel. (b) Purified MTIP-His by Ni-NTA Column chromatography. Commassie stained 15% polyacrylamide gel. Lane M: Marker, Lane 1: Purified MTIP. (17.4 kDa). Western blot of purified MTIP-His using anti-MTIP.

Figure S2: Negative control for ELISA and ITC experiments. (a) 500 ng non-specific *Mycobacterial* protein ESAT6 was coated on ELISA plates and indicated concentrations of ZA1 and MTIP were overlaid respectively. No interaction was observed between the coated and overlaid proteins. (b) The raw calorimetric data denoting the amount of heat produced following each injection of Myosin A from syringe into cell containing buffer only. No heat changes were observed when Myosin A was added into buffer. (c) Heat changes for competitive binding when pre-bound Myosin A-ZA1 complex is titrated from syringe into MTIP in the cell. No heat changes are observed denoting that Myosin A-ZA1 complex does not break when added into MTIP.

Figure S3:

(a)

**ZA1**	**SGRHVFKQLDIDIDIMHELDIELELDIDIDIMHPGDIDIDIDIDIQLDIELDIMHDIDIDIMHMHQLELELDIFKHVAAA**
**ZA2**	**SGRHVFKPGMHELDIELELDIELDIDIDIELDIDIELDIDIDIDIQLYVDIELMHELDIDIRTELELDIPGELEFDIELDIDIFKHVAAA**
**ZA3**	**SGRHVFKELDIELDIDIDIDIDIDIDIELMHDIQLDIELDIDIMHMHDIPGDIDIMHSTMHQLDIELMHPGDIDIQLFKHVAAA**
**ZA4**	**SGRHVFKQLDIDIDIMHELDIELELDIDIDIMHPGDIDIDIDIDIQLDIELDIMHDIDIDIMHMHQLELELDIFKHVAAA**

(b)

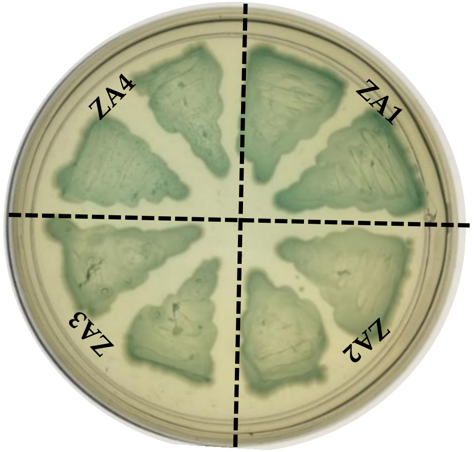

Figure S3: Myosin A tail binders from de novo peptide library. (a) Sequence of peptide binders against Myosin A tail. Sequence of ZA1 and ZA4 were found to be identical. (b) Liquid patching of the blue colonies obtained upon screening of Myosin A tail with DIEL library.

Figure S4:

(a) (b)

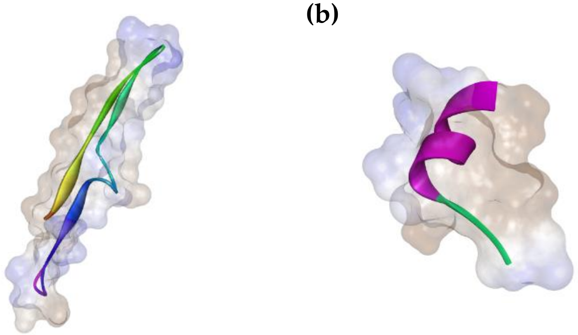

Figure S4: Prediction Models. (a) Myosin A tail/ZA1 and (b) Myosin A tail/ZA1S surface model used for Ramachandran Analysis

Figure S5:

(a)

>XP_001350849.1 Myosin A Tail Domain Interacting Protein [Plasmodium falciparum 3D7]

MKQECNVCYFNLPDPESTLGPYDNELNYFTWGPGFEYEPEPQRKPLSIEESFENSEESEESVADIQQLEEKVDESDVRIYFNEKSSGGKISIDNASYNARKLGLAPSSIDEKKIKEL
YGDNLTYEQYLEYLSICVHDKDNVEELIKMFAHFDNNCTGYLTKSQMKNILTTWGDALTDQEAIDALNAFSSEDNIDYKLFCEDILQ

(b)

>XP_001350147.1 Myosin A [Plasmodium falciparum 3D7]

MAVTNEEIKTASKIVRRVSNVEAFDKSGSVFKGYQIWTDISPTIENDPNIMFVKCVVQQGSKKEKLTVVQIDPPGTGTPYDIDPTHAWNCNSQVDPMSFGDIGLLNHTNIPC
VLDFLKHRYLKNQIYTTAVPLIVAINPYKDLGNTTNEWIRRYRDTADHTKLPPHVFTCAREALSNLHGVNKSQTIIVSGESGAGKTEATKQIMRYFASSKSGNMDLRIQTAI
MAANPVLEAFGNAKTIRNNNSSRFGRFMQLVISHEGGIRYGSVVAFLLEKSRIITQDDNERSYHIFYQFLKGANSTMKSKFGLKGVTEYKLLNPNSTEVSGVDDVKDFEEVIE
SLKNMELSESDIEVIFSIVAGILTLGNVRLIEKQEAGLSDAAAIMDEDMGVFNKACELMYLDPELIKREILIKVTVAGGTKIEGRWNKNDAEVLKSSLCKAMYEKLFLWIIRHL
NSRIEPEGGFKTFMGMLDIFGFEVFKNNSLEQLFINITNEMLQKNFVDIVFERESKLYKDEGISTAELKYTSNKEVINVLCEKGKSVLSYLEDQCLAPGGTDEKFVSSCATNLKE
NNKFTPAKVASNKNFIIQHTIGPIQYCAESFLLKNKDVLRGDLVEVIKDSPNPIVQQLFEGQVIEKGKIAKGSLIGSQFLNQLTSLMNLINSTEPHFIRCIKPNENKKPLEWCEP
KILIQLHALSILEALVLRQLGYSYRRTFEEFLYQYKFVDIAAAEDSSVENQNKCVNILKLSGLSESMYKIGKSMVFLKQEGAKILTKIQREKLVEWENCVSVIEAAILKHKYKQK
VNKNIPSLLRVQAHIRKKMVAQ

Figure S5: Sequences of MTIP and Myosin A. Complete sequence of *Pf*MTIP 204 amino acids (a) and *Pf*Myosin A 818 amino acids long (b). Sequence in red indicates the interacting regions of the two proteins that are used for the present study.
